# The Effect of p38MAPK on Cyclic Stretch in Human Facial Hypertrophic Scar Fibroblast Differentiation

**DOI:** 10.1371/journal.pone.0075635

**Published:** 2013-10-09

**Authors:** Qi-cui Du, Dai-zun Zhang, Xiu-juan Chen, Gui Lan-Sun, Min Wu, Wen-lin Xiao

**Affiliations:** 1 Department of Stomatology, The Affiliated Hospital of Medical College, Qingdao University, Qingdao, the People’s Republic of China; 2 Department of Paediatric Dentistry, The Affiliated Hospital of Medical College, Qingdao University; Qingdao, the People’s Republic of China; 3 Department of Biochemistry & Molecular Biology, University of North Dakota, Grand Forks, North Dakota, United States of America; Universidade Federal do Rio de Janeiro, Brazil

## Abstract

Hypertrophic scars (HTS), the excessive deposition of scar tissue by fibroblasts, is one of the most common skin disorders. Fibroblasts derived from surgical scar tissue produce high levels of α-smooth muscle actin (α-SMA) and transforming growth factor-β1 (TGF-β1). However, the molecular mechanisms for this phenomenon is poorly understood. Thus, the purpose of this study was to evaluate the molecular mechanisms of HTS and their potential therapeutic implications. Fibroblasts derived from skin HTS were cultured and characterized *in vitro*. The fibroblasts were synchronized and randomly assigned to two groups: cyclic stretch and cyclic stretch pre-treated with SB203580 (a p38MAPK inhibitor). Cyclic stretch at 10% strain was applied at a loading frequency of 10 cycles per minute (i.e. 5 seconds of tension and 5 seconds of relaxation) for 0 h, 6 h and 12 h. Cyclic stretch on HTS fibroblasts led to an increase in the expression of α-SMA and TGF-β1 mRNA and protein and the phosphorylation of p38MAPK. SB203580 reversed these effects and caused a decrease in matrix contraction. Furthermore, HTS fibroblast growth was partially blocked by p38MAPK inhibition. Therefore, the mechanism of cyclic stretch involves p38 MAPK, and its inhibition is suggested as a novel therapeutic strategy for HTS.

## Introduction

Hypertrophic scars (HTS) are a common clinical pathological state. They are characterized by the proliferation of a large number of fibroblasts, excessive deposition of extracellular matrix (ECM) and microvascular dysplasia with infiltration of inflammatory cells [1]. Histologically, HTS show excess accumulation of collagen fiber bundles, with a high density of endothelial cells, fibroblasts and myofibroblasts (MFB) [2]. Despite advances in our knowledge about the molecular pathogenesis of HTS, their mechanism is not yet fully understood [3]. A constituent of HTS is the invasive growth of fibroblasts; thus, HTS can be a major problem in plastic surgery [4]. Fibroblasts are an important type of cells that are related to fibrillogenesis and wound healing. In scarless or regenerative healing, fibroblasts are quiescent and do not significantly proliferate, but provide enough ECM synthesis to maintain adequate tissue strength. By contrast, fibroblast activation leads to cell proliferation and ECM synthesis, reducing collagen remodeling.

MFB, the primary source of ECM contraction, are characterized by an increase in α-SMA and TGF-β1 [5]. During the progression of HTS, fibroblasts are activated and transdifferentiated into MFB. Indeed, the fibroblasts that have been transformed to MFB express a much greater level of α-SMA than the parental fibroblasts [6]. Thus, α-SMA has been established as a representative marker of MFB [7]. In the process of fibrosis, a large quantity of ECM is synthesized by the MFB and deposited in the tissues and organs that are involved, resulting in structural reconstruction of the junction and functional abnormalities [8].

TGF-β1 is believed to play an important role in wound healing and HTS formation [9]. TGF-β1 is a potent regulator of cellular proliferation and ECM interaction, although wound healing and scar formation are also regulated by other growth factors and cytokines [10], [11]. To date, researchers have confirmed that TGF-β1 can lead normal fibroblasts to transdifferentiate to MFB [12]. TGF-β1 regulates Smad, an intermediary signaling molecule. Cyclic stretch sends signals from the cell membrane into the nucleus to activate the transcription of target genes, resulting in the activation of the TGF-β1/Smad signal transduction pathway [13]. TGF-β1 can regulate the epithelial mesenchymal transition via the Smad signaling pathway in various tissues, including the skin, lungs, kidneys and other tissues [14]. In recent years, researchers have found that some of the non-Smad pathways, such as the mitogen activated protein kinase (MAPK) pathway, may also play a key role in signal transduction. Currently, the MAPK pathway can be categorized into four pathways [15]. Among these, p38MAPK is involved in the process of cell transdifferentiation [16]. P38MAPK is also an important signal of mechanical transmission. Under cyclic stretch, p38MAPK may affect the transformation of MFB.

Normal wound healing involves a complex interaction between many cell types and the microenvironment [17]. For children with cleft lip and palate, surgical repair may be performed to correct the defect, but the scar formation affects facial appearance and function. The patients usually have to suffer additional operations to remove the facial scar. However, the wound healing process is influenced by multiple factors that compose a complex process. One such factor is mechanotransduction, the cellular event whereby mechanical stimuli are converted into biochemical signals. *In vivo*, cells may experience mechanical forces through the pulsatile nature of blood flow or during the process of wound contraction [18], [19]. Fibroblasts play a key role in granulation tissue formation during the proliferative phase of wound healing. During this phase, fibroblasts migrate into the wound bed to proliferate and synthesize growth factors. In addition, the production of new ECM and other proteins, such as actin, is essential to wound healing [20]. In two-dimensional cell culture, application of mechanical force in the form of cyclic strain regimes of 6–120 cycles per minute (cpm) has been confirmed to influence many aspects of fibroblast physiology, including migration, proliferation, protein synthesis and gene expression [21], [22]. Furthermore, studies have shown that various Rho family members can activate other targets, such as those in the p38MAPK family [23]. In the current study, we examined the *in vitro* effect of cyclic stretch on the p38MAPK signal transduction pathway and transdifferentiation of fibroblasts derived from primary human HTS. Our results demonstrate that p38MAPK is involved in the activation of fibroblasts under cyclic stretch and that specific inhibition of p38MAPK blocks this effect.

## Materials and Methods

### Patient Inclusion Criteria

HTS patients were selected according to the Vancouver Scar Scale (VSS) and ranged from a score of 10 to 13 [24]. HTS tissues were obtained from 10 people (7 males and 3 females, ranging in age from 6 to 38 years; mean age: 22 years), who received scar resection 6–12 months following cleft lip and palate surgery. Clinical manifestations of the HTS included scars that exhibited obvious hyperemia, were red in appearance and had obvious hypertrophy. Patients also exhibited pruritus, pain and synesthesia and the scars were all confirmed pathologically to be in the proliferative phase. There were no local infections or ulcerations present and no cases were treated with glucocorticosteroids or radiotherapy. Prior to surgery, all patients were informed of the purpose and procedure of this study and agreed to the collection of their tissue specimens. Written consent was obtained from all participants involved in this study. The Ethic Committee of The Affiliated Hospital of Qingdao University, Qingdao, China approved all protocols.

### Preparation of Scar Specimens

All HTS specimens were obtained from patients who underwent cleft lip and palate surgery at the Institute of Oral and Maxillofacial Surgery of Affiliated Hospital of Qingdao University. The specimens were washed with phosphate buffered saline (PBS) and subjected to immediate cell isolation or fixed with saturated trinitrophenol solution and stored in liquid nitrogen.

Because patient tissues are difficult to obtain, the HTS specimen of each patient was first collected and stored in liquid nitrogen. After all the 10 patients’ HTS specimens were collected, the specimens were thawed for primary cell culture to harvest enough cells to carry out subsequent experiments. Three independent Western blotting and RT-PCR experiments were performed (N = 3).

### Primary Culture and Passage

For sterilization, each skin piece was soaked in Mg- and Ca-free PBS, pH 7.4, supplemented with penicillin G potassium (100 U/ml) and streptomycin sulfate (0.1 mg/ml). As described previously [25], HTS tissue was cut into 0.3–0.5 mm pieces, and the epidermis and dermis were isolated by digestion with 0.25 g/l dispase II. The dermal tissue was minced and digested thoroughly with 30 volumes of 200 U/ml collagenase I solution in PBS at 37°C for 2 hours, followed by centrifugation and collection of cells.

In the course of primary cell culture, the cells were cultured in DMEM medium, containing 15% fetal calf serum (FCS), at 37°C in 5% CO_2_. The culture medium was changed every 3 days. Cell morphology and growth were observed under an inverted microscope. At 80%–90% confluency, the medium was aspirated, and the cells were washed three times with PBS. The samples were then digested with pancreatin for 1.0 to 3.0 minutes. Cells were observed under the inverted microscope. When their appearance changed from spindle-shaped to round, an equal volume of medium was added to terminate the digestion. Cells on the wall of the culture flask were triturated using a pipette. The cell suspension was centrifuged at 1000 rpm (200 g) for 5 minutes. Supernatant was removed, adequate growth medium was added and the cells were triturated. The cell suspension was placed in a new culture flask and treated with growth medium for further culture. Cells were passaged 3 to 5 times and used directly in experiments.

### Immunostaining

For indirect immunofluorescence, the fibroblasts were plated at a density of 2×10^4^ per well in 24-well plates overnight and were washed three times with PBS (5 minutes each wash). After 20 minutes, the cells were fixed with 4% paraformaldehyde, permeabilized with 0.3% Triton-100 for 10 min and then blocked with 10% goat serum (at 37°C in a humidified incubator for 20 min). Primary vimentin antibody (Goat Anti-Rabbit IgG, 1∶100) was added, and the cells were placed at 4°C overnight. The following day, the cells were treated with secondary antibody (Rabbit Anti-goat IgG, 1∶200) and placed in a 37°C incubator for 60 min in the dark. Imaging of the immunofluorescence staining was captured using a Laser Confocal Scanning Microscope (LCSM). The results were observed under 488 nm emissions.

### Establishment of Mechanical Stimulation Models

Cells were seeded into flexible wells and subjected to periodic negative pressure using the Flexcell vacuum instrument (X-4000, Flexcell International Corporation, Hillsborough, NC). Because of the design of the flexible culture wells, cells grow under constant tensile stress, which varies depending on the seeding position. The center areas of the wells have essentially no tensile stress while the peripheral areas have maximal tensile stress due to a gradual increase in stress from the center to the edge of the wells [26].

Cells at 1×10^5^/L were seeded in eight Bioflex 6-well culture plates coated with Poly-L-Lysine Solution (2 ml/well). The medium was replaced on alternate days. At 80%–90% confluency, the medium was replaced and serum-free medium was added for 24 hours to synchronize the cells. Cells were randomly assigned to control, loading and SB203580 groups. In the loading and SB203580 groups, fibroblasts on the 6-well elastic basal membrane culture plate were loaded using an FX-4000T charger. A 10% strain was applied at a loading frequency of 6 cycles per minute (*i.e*. 5 seconds for tension and 5 seconds for relaxation in each cycle). In the SB203580 group, cells were treated with 20 mmol/L of the p38MAPK inhibitor, SB203580, 1 hour before loading. HTS fibroblasts from each group were harvested at 0 h, 6 h and 12 h after loading.

### Western Blot Analysis

Western blot analysis was performed to measure the protein expression of p-p38MAPK, TGF-β1 and α-SMA. Cells were rinsed with ice-cold PBS, and cell lysates were prepared by treating the fibroblasts with RIPA lysis buffer (1×PBS, 1% Nonidet P-40, 0.5% sodium deoxycholate and 0.1% SDS) plus protease inhibitors (0.1% phenylmethylsulfonyl fluoride; PMSF) for 30–60 min. The lysates were sonicated 3 times for 5 s on ice and were then centrifuged at 10,000×g for 10 min (4°C) to precipitate the particulate material. Protein concentrations were measured with a BCA assay. The proteins were resolved by sodium dodecyl sulfate- polyacrylamide gel electrophoresis and transferred onto a polyvinylidene difluoride membrane using a gel blot apparatus. Blots were blocked with 5% nonfat dry milk in PBST (17 mM KH_2_PO_4_, 50 mM Na_2_HPO_4_, 1.5 mM NaCl, pH 7.4, and 0.05% Tween-20) for 1 h at room temperature and then were incubated with primary antibody overnight at 4°C. Proteins were then incubated with a peroxidase-conjugated secondary antibody for 45 minutes at room temperature. After each incubation step, the membranes were washed in TBST for 20 minutes. Protein bands were detected, and the relative intensities of the protein bands were analyzed by autoradiography using Quantity One software to calculate ratios of expression of the proteins of interest standardized to β-actin expression. Averages and SD were calculated for the ratios in three independent experiments using SPSS 17.0 software. The antibodies used in the present study were P-p38 kinase antibody (1∶4,000, Cell Signaling Technology, Boston, MA), TGF-β1 antibody (1∶4,000, Cell Signaling Technology, Boston, MA), α-SMA antibody (1∶4,000, Abcam, Cambridge, MA) and β-actin (1∶10,000, Cell Signaling Technology, Boston, MA).

### RNA Isolation and Real-time RT-PCR

Harvested HTS fibroblasts from each group were treated with the cell-specific p38MAPK inhibitor, SB203580 for different lengths of time and were then collected. After experimental treatments, the plates were placed on ice, and the cells were washed twice with cold PBS at pH 7.4. Cells from as many as ten wells were pooled, and total RNA was isolated by the guanidinium thiocyanate-phenol-chloroform method of Chomczynski and Sacchi [27]. RNA concentrations were determined by measuring the optical density at 260 nm (OD260), and purity was estimated by determining the OD260/OD280 ratio. The cDNA fragment of the target gene was amplified using a reverse transcription-polymerase chain reaction. Target gene expression in fibroblasts was detected using a semiquantitative polymerase chain reaction. The primers were designed by Premier 5.0 and synthesized by Shanghai Shenggong Genomics Institute. Primer sequences are shown in Table 1. Polymerase chain reaction products were identified using 2.0% agarose gel electrophoresis and then photographed. Experiments were repeated three times, each in triplicate.

**Table 1:** Sequences of α-SMA, TGF-β1 and GAPDH primers.

Primer	Sequence (5′-3′)	Size (bp)
α-SMA	Upstream: CTGAAGAGCATCCCACCCTG Downstream: TGCGTCCAGAGGCATAGAGA	150
TGF-β1	Upstream: GAGAAGCGGTACCTGAACCC Downstream: GGTTGCTGAGGTATCGCCAG	132
GAPDH	Upstream: TCATGGGTGTGAACCATGAGAA Downstream: GGCATGGACTGTGGTCATGAG	146

### Statistical Analysis

The data are expressed as mean ± SD and were analyzed using SPSS 17.0 software. One-way ANOVA (for the loading group) and independent-sample T Tests (for the SB203580 group vs. the loading group) were used for intragroup comparisons, and the least-significant difference was used for intergroup comparisons. A value of *P*<0.05 was considered statistically significant.

## Results

### Morphological Observation and Identification of Primary Cultured HTS Fibroblasts

**Figure 1:**
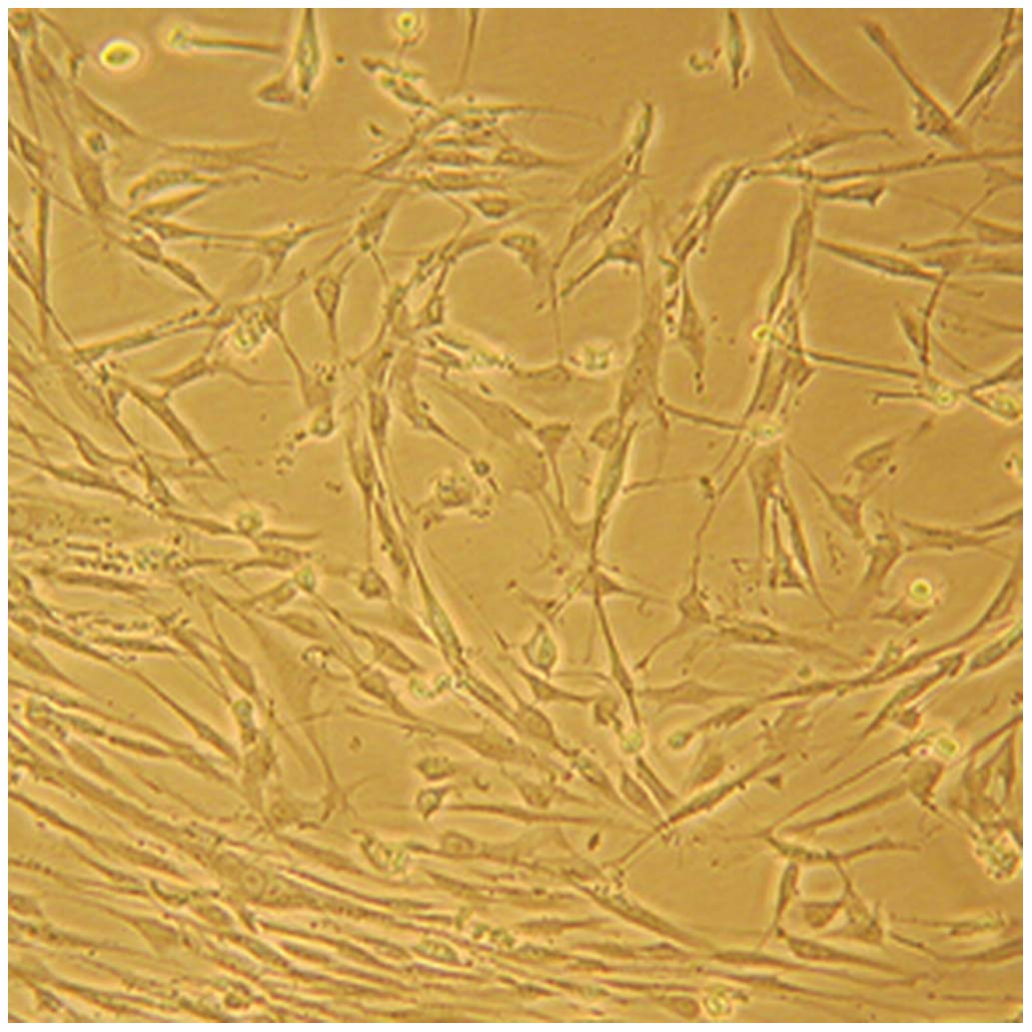
Microscopic observation of HTS fibroblasts. A representative image is shown of primary cultured HTS fibroblasts (400× magnification). The cells appeared elongated or flattened, and some were triangular. Cytoplasm extended outward 2–3 lengths of various different tubers. In the process of growth, cells displayed radial, braided or spiral arrangement.

To confirm the establishment of a third generation of primary cultured HTS fibroblasts, the cells were observed microscopically as shown in Figure 1. The confirmed morphological features of fibroblasts, such as elongated or flattened cell bodies with some parts being triangular and with radial or spiral arrangements, were observed.

### Verification of HTS Fibroblastic Morphology by Immunofluorescence Analysis

**Figure 2:**
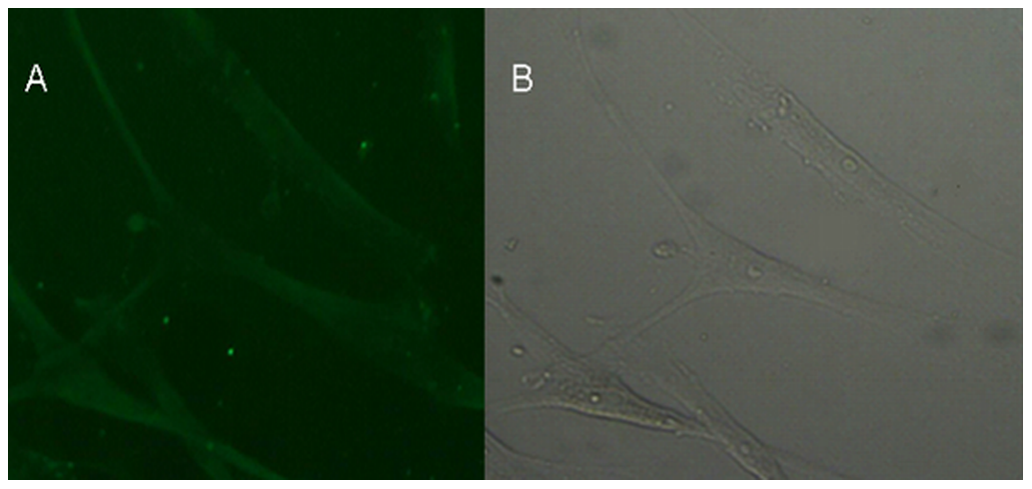
Immunofluorescent identification of HTS fibroblasts. **A**. Representative image of HTS fibroblast following immunofluorescence staining with vimentin antibody. The presence of vimentin was observed to confirm the identification of scar fibroblasts (10× magnification). **B**. Corresponding immunofluorescence of normal microscopic scar fibroblasts is shown (10× magnification). Immunofluorescent staining indicated that the fibroblasts expressed the characteristic marker vimentin. Hyperplastic scar fibroblasts have ordinary fibroblast characteristics.

The cells were fixed and stained with vimentin antibody for the detection of fibroblasts. Immunofluorescence detection of vimentin fibroblasts showed brown granules and green cytoplasm; however there was no significant staining of the nuclei (Figure 2). Fig. 2A shows a representative image from immunofluorescence microscopy of HTS fibroblasts identified by vimentin expression. Fig. 2B represents the corresponding immunofluorescence image of normal microscopic scar fibroblasts.

### Cyclic Stretch Enhances the Migration of Human HTS Fibroblasts

**Figure 3:**
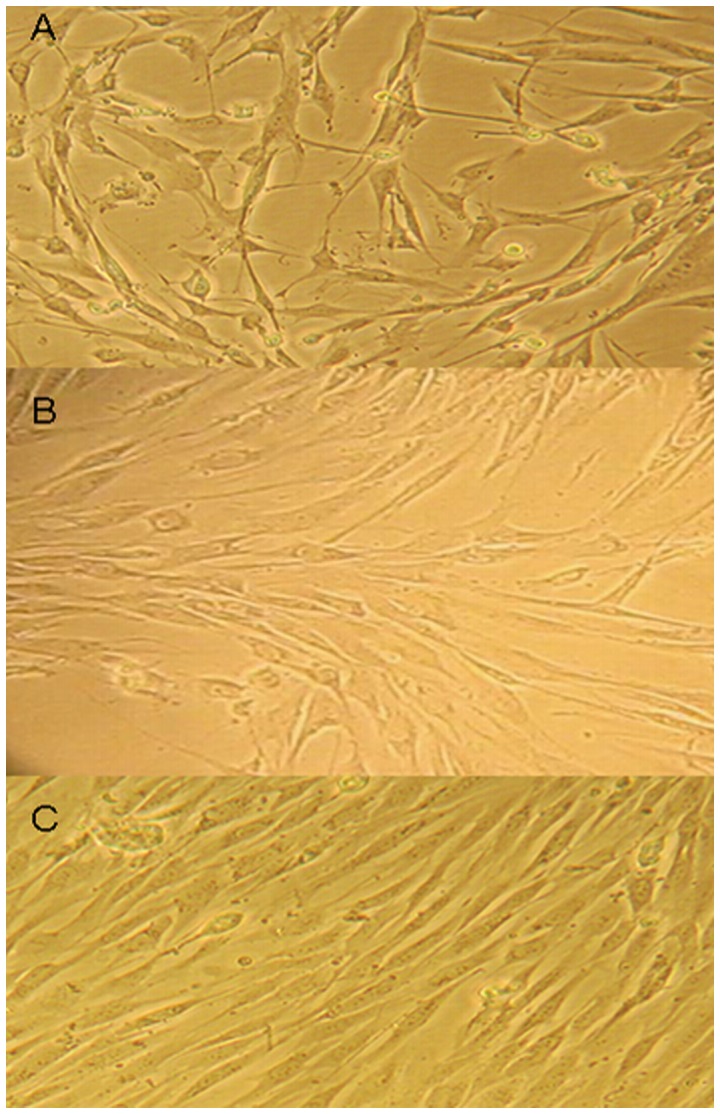
Cyclic stretch impacts HTS fibroblast morphology. **A**. HTS fibroblast loading 0 h strength (400×). Cell morphology and direction of growth did not change. **B**. HTS fibroblasts loading 6 h strength (400×). The direction of cell growth started to change and complied with the direction of the cyclic stretch. **C.** HTS fibroblasts loading 12 h strength (400×). The direction of cell growth obviously complied with the direction of the cyclic stretch. The results show that the characteristics of fibroblasts changed with the increase in cyclic stretch time.

At 0 h loading, the cell morphology and direction of growth did not change (Fig. 3A). However, at 6 h loading, the direction of cell growth began to change according to the direction of the cyclic stretch (Fig. 3B). This feature became even more obvious after 12 h loading (Fig. 3C). These results demonstrate that continuous cyclic stretch induces responses in fibroblasts. Furthermore, the degree of morphological change in the cells, e.g., the deformation of the cells, was associated with the duration of the cyclic stretch. This data confirm that cyclic stretch has the ability to increase the formation of scars.

### Cyclic Stretch Enhances the Levels of Phosphorylated p38MAPK in Human HTS Fibroblasts

**Figure 4:**
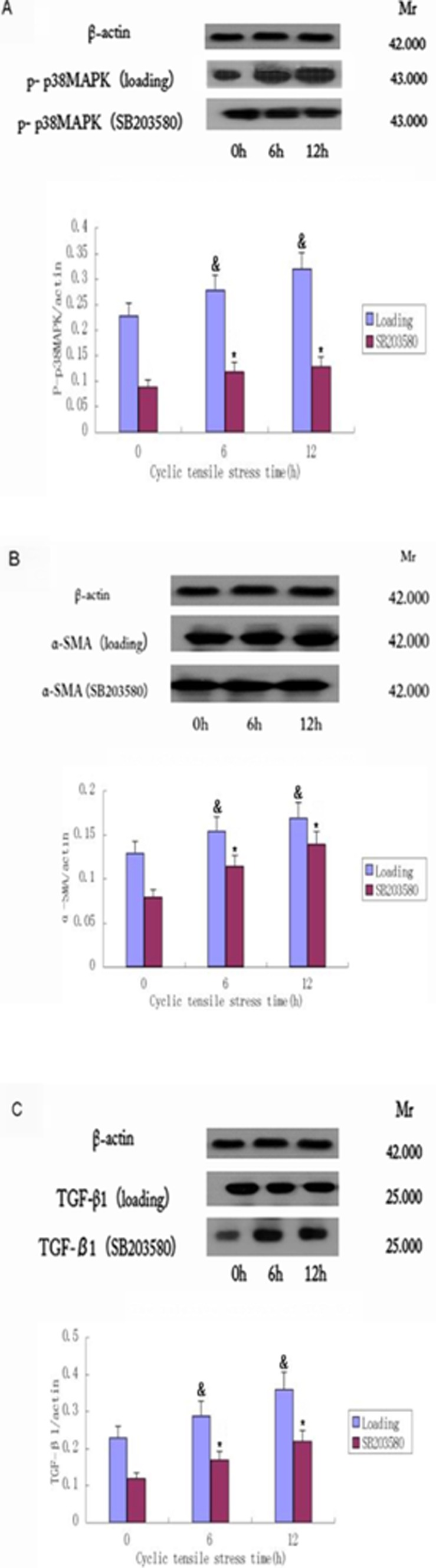
Cyclic stretch results in decreased expression of P-p38MAPK, α-SMA and TGF-β1. Western blotting was performed to assess changes in the expression levels of (A) P-p38MAPK, (B) α-SMA and (C) TGF-β1 and after 0 h, 6 h or 12 h cyclic stretch (loading). The role of p38MAPK was also assessed by the same cyclic stretch administration after pretreatment with the p38MAPK inhibitor SB203580 (SB203580). A representative image for each experiment is shown, with means +/− SD of three independent experiments graphed below each set of images. The expression of all three proteins were significantly induced by cyclic stretch and suppressed by SB203580. The levels of α-SMA, TGF-β1 and P- p38MAPK and β-actin in fibroblasts were quantified by densitometry analysis using Quantity One software. The expression of each protein band was standardized to β-actin expression, and means and SD of the ratios in triplicate experiments were calculated using SPSS 17.0 software. ^&^, p<0.05 vs. the corresponding time 0 in the loading group; *, p<0.05 for loading vs. SB203580 (for 6 h and 12 h timepoints only). A full set of each of the three replicates of this experiment are provided in Figure S1, and a complete set of data are provided in Tables S1, S2, S3.

MAPK is thought to be an important signal that predicts the strength of the stretch in eukaryotes. To assess whether heightened p38MAPK activity contributes to the enhanced fibrotic phenotype of HTS, we used Western blotting to analyze the expression of P-p38MAPK in human HTS fibroblasts. Levels of P-p38MAPK were elevated following 6 h and 12 h cyclic stretch. As a control, cells incubated for 12 h with the p38MAPK inhibitor SB203580 (20 mM) showed reduced P-p38MAPK activation (Figure 4A and Table S1). These results demonstrate that p38MAPK is activated during cyclic stress in HTS and verify the function of SB203580 in preventing this activation.

### Cyclic Stretch Directly Promotes a Fibrogenic Phenotype in Human HTS Fibroblasts via P38MAPK

To test whether cyclic stretch promotes a fibrogenic phenotype in HTS, we assessed the induction of the fibrogenic markers α-SMA and TGF-β1. Western blotting demonstrated that both proteins are induced during cyclic stretch (Figure 4B and C; Tables S2 and S3). Furthermore, SB203580 inhibitor treatment decreased the expression of both α-SMA and TGF-β1, suggesting that their expression is regulated by p38MAPK.

**Figure 5:**
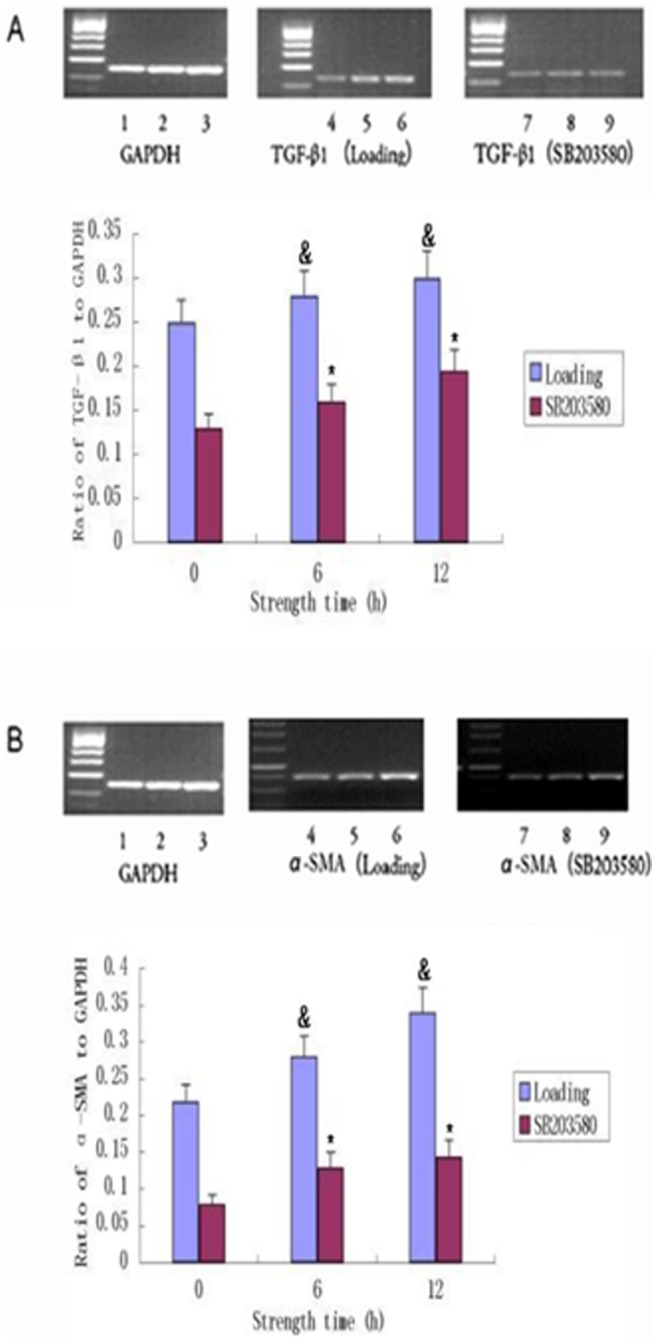
Effects cyclic stretch on *α-SMA* and *TGF-β1* mRNA expression. Primary cultures of fibroblasts (1×10^6^ cells) were exposed to cyclic stretch for 0 h, 6 h and 12 h without (loading) or with p38MAPK inhibitor (SB203680). Real-Time PCR results demonstrated that *α-SMA* and *TGF-β1* mRNA expression was effectively suppressed by 6 h and 12 h cyclic stretch. The left panels show the results of a representative experiment, and the right panels show the means ± SD of densitometric values from three independent experiments. Lanes 1,2.3: strength at 0 h, 6 h and 12 h in GAPDK; 4, 5, 6: strength at 0 h, 6 h and 12 h in the loading group; Lanes 7, 8, 9: strength at 0 h, 6 h and 12 h in the SB203580 group. ^&^, p<0.05 vs. time 0. *, p<0.05 for loading versus SB203580 (for 6 h and 12 h timepoints only). Data are representative of three experiments.

To validate the protein data and to determine whether the expression of α-SMA and TGF-β1 is regulated at the level of transcription, we assessed the expression of *TGF-β1 and α-SMA* mRNA following cyclic stretch. Compared with the control group, the levels of TGF-β1 mRNA and α-SMA mRNA were significantly greater in the stretch-loaded group at 12 h, while the changes at 6 h were less dramatic (Figure 5). These results verify that cyclic stretch induces the transdifferentiation of fibroblasts and suggest that the effect is more significant with longer periods of loading. Consistent with their regulation by p38MAPK, the levels of *TGF-β1* and *α-SMA* mRNA were diminished in the SB203580 group compared to the control group with stretch loading (*P*<0.05) (Figure 5), thus confirming our Western studies suggesting that cyclic stretch has the capacity to promote the fibrogenic phenotype through p38MAPK.

## Discussion

HTS forms erythematous scar tissue with pruritis. The inelastic qualities of the scar and the frequent occurrence of contractures may severely restrict the mobility (especially for the joints and extremities), and can immobilize structures, such as eyelids [28]. Treatment for HTS is problematic, with no single modality having uniformly satisfactory results.

MFBs are responsible for the excessive deposition and irreversible remodeling of the ECM, which is a hallmark of virtually all fibrotic diseases and impedes organ function, often leading to lethal organ failure [29]. Transformation from HTS fibroblasts to MFBs is an important step in wound healing. During MFB differentiation, fibroblastic cells lose their migratory phenotype and become sessile. This switch is promoted by the formation of large focal adhesions that enable strong attachments between intracellular stress fibers and ECM proteins, such as collagen and fibronectin [30]. Strong contraction and adhesion enable the MFB to remodel tissues, even against the considerable stress arising during tissue remodeling [31], [32]. Previous studies have shown that scar fibroblast differentiation relies on α-SMA, TGF-β1 and stress. Transformation from HTS fibroblasts to MFB occurs in response to TGF-β1 and heightens tension in the extracellular environment [33]. MAPK, a class of highly conserved serine protein kinase within the cytoplasm [34], is part of an important biological signal transduction system which participates in the mediation of cell growth, division, differentiation and death, as well as the synchronous function between cells.

The present study was done to explore the effects of cyclic stretch on the p38MAPK signal transduction pathway and fibroblast transdifferentiation in primary human HTS fibroblasts *in vitro*. Our results demonstrate that cyclic stretch can lead scar fibroblasts to undergo MFB differentiation. Cyclic stretch increased the expression of α-SMA, TGF-β1 and p38MAPK in scar fibroblasts. In addition, we also demonstrate that with increased strength, the expression of α-SMA, TGF-β1 and p38MAPK was also increased. These changes were statistically significant (Figure 4 and Figure 5) and suggest a role for p38MAPK in the induction of α-SMA and TGF-β1. The increase in the expression of α-SMA is consistent with the transdifferentiation of scar fibroblasts to MFB in response to cyclic stretch. Additionally, TGF-β1 is a multifunctional growth factor that plays a critical role in cell function, epithelial cell growth and ECM deposition. Overproduction of TGF-β1 is associated with excessive deposition of scar tissue and fibrosis [35]. TGF-β1 can also activate p38MAPK [36], as demonstrated in our previous study of human dermal fibroblasts [37]. P38MAPK is a positive regulator of collagen synthesis in dermal fibroblasts and a mediator of TGF-β1, stimulating ECM production. In human dermal fibroblasts, TGF-β1 also has been shown to transduce signals through the p38MAPK pathway to stimulate collagen production [38]. The MAPK signaling pathway is induced by TGF-β1 and can modulate the outcome of the TGF-β1-induced responses [39]. In particular, p38MAPK has been shown to mediate Smad-independent TGF-β1 responses [40]. Thus, we can speculate that TGF-β1 and p38MAPK may be involved in cross-talk.

Our results show that inhibition of p38MAPK, specifically by the inhibitor SB203580, interferes with the stimulatory effects of cyclic stress on the production of ECM proteins. These findings suggest that stimulation of ECM production by cyclic strength likely results from cooperative effects of multiple signaling including p38MAPK, leading to scar fibroblast transdifferentiation to MFB. Nevertheless, the exact mechanism that mediates the action of p38MAPK on the fibrogenic response has not yet been identified. Using the p38MAPK inhibitor, our experimental results showed that α-SMA and TGF-β1 induction were reduced. Therefore, p38MAPK affected TGF-β1 to a certain extent. p38MAPK has also been reported to be directly required for TGF-β1-dependent stimulation of matrix production by dermal fibroblasts [41], [42], [43]. Thus, the inhibition of p38MAPK may potentially be effective in preventing dermal scarring.

The biggest obstacle in the investigation of skin fibrosis is the lack of animal models and trusted human pathological samples [44], [45]. These specimens represent the final process of scar formation and can be assessed during scar formation for initial impact factors. Thus, primary cultured hyperplastic scar fibroblasts were used to reflect the *in vivo* environment.

In summary, we demonstrated that cyclic stretch in human HTS fibroblasts can affect the p38MAPK signal transduction pathway and transdifferentiation of fibroblasts *in vitro*. In the process of wound healing, tension is unavoidable. However, these studies suggest that we might consider blocking p38MAPK clinically in the wound healing process in order to reduce scar formation.

## Supporting Information

Table S1 **Summary of P-p38MAPK Western data from three experimental replicates.**
(DOCX)

Table S2 **Summary of α-SMA Western data from three experimental replicates.**
(DOCX)

Table S3 **Summary of TGF-β1 Western data from three experimental replicates.**
(DOCX)
