# Supplementary material for: The Effect of p38MAPK on Cyclic Stretch in Human Facial Hypertrophic Scar Fibroblast Differentiation
Source: PLoS One. 2013 Oct 9;8(10):e75635. doi: 10.1371/journal.pone.0075635 (PMC3794006; doi:10.1371/journal.pone.0075635)
Supplement: Table S1 — Summary of P-p38MAPK Western data from three experimental replicates. (DOCX) [file pone.0075635.s001.docx]

**Table S1**

| **P-p38MAPK** |  | **0h** | **6h** | **12h** |
| --- | --- | --- | --- | --- |
| **Loading group** | **Mean** | 0.239 | 0.288 | 0.320 |
|  | **Standard deviation** | 0.015 | 0.016 | 0.017 |
|  | **P value vs 0h** |  | 0.000 | 0.000 |
|  |  |  |  |  |
| **SB203580 group** | **Mean** | 0.087 | 0.128 | 0.130 |
|  | **Standard deviation** | 0.015 | 0.016 | 0.017 |
|  | **P value vs 0h** |  | 0.000 | 0.000 |
|  | **P value vs Loading** |  | 0.008 | 0.006 |
