# Supplementary material for: The Effect of p38MAPK on Cyclic Stretch in Human Facial Hypertrophic Scar Fibroblast Differentiation
Source: PLoS One. 2013 Oct 9;8(10):e75635. doi: 10.1371/journal.pone.0075635 (PMC3794006; doi:10.1371/journal.pone.0075635)
Supplement: Table S2 — Summary of α-SMA Western data from three experimental replicates. (DOCX) [file pone.0075635.s002.docx]

**Table S2**

| **α-SMA** |  | **0h** | **6h** | **12h** |
| --- | --- | --- | --- | --- |
| **Loading group** | **Mean** | 0.134 | 0.152 | 0.172 |
|  | **Standard deviation** | 0.011 | 0.014 | 0.017 |
|  | **P value vs 0h** |  | 0.000 | 0.000 |
|  |  |  |  |  |
| **SB203580 group** | **Mean** | 0.083 | 0.116 | 0.150 |
|  | **Standard deviation** | 0.011 | 0.018 | 0.013 |
|  | **P value vs 0h** |  | 0.000 | 0.000 |
|  | **P value vs Loading** |  | 0.033 | 0.018 |
