# Supplementary material for: The Effect of p38MAPK on Cyclic Stretch in Human Facial Hypertrophic Scar Fibroblast Differentiation
Source: PLoS One. 2013 Oct 9;8(10):e75635. doi: 10.1371/journal.pone.0075635 (PMC3794006; doi:10.1371/journal.pone.0075635)
Supplement: Table S3 — Summary of TGF-β1 Western data from three experimental replicates. (DOCX) [file pone.0075635.s003.docx]

**Table S3**

| **TGF-β** |  | **0h** | **6h** | **12h** |
| --- | --- | --- | --- | --- |
| **Loading group** | **Mean** | 0.223 | 0.300 | 0.369 |
|  | **Standard deviation** | 0.012 | 0.012 | 0.015 |
|  | **P value vs 0h** |  | 0.000 | 0.000 |
|  |  |  |  |  |
| **SB203580 group** | **Mean** | 0.128 | 0.181 | 0.228 |
|  | **Standard deviation** | 0.013 | 0.018 | 0.019 |
|  | **P value vs 0h** |  | 0.000 | 0.000 |
|  | **P value vs Loading** |  | 0.007 | 0.005 |
